# Knockout Gene-Based Evidence for PIWI-Interacting RNA Pathway in Mammals

**DOI:** 10.3389/fcell.2021.681188

**Published:** 2021-07-14

**Authors:** Yinuo Li, Yue Zhang, Mingxi Liu

**Affiliations:** ^1^State Key Laboratory of Reproductive Medicine, Department of Histology and Embryology, School of Basic Medical Sciences, Nanjing Medical University, Nanjing, China; ^2^State Key Laboratory of Reproductive Medicine, Clinical Center of Reproductive Medicine, The First Affiliated Hospital of Nanjing Medical University, Nanjing, China

**Keywords:** piRNA, meiosis, male infertility, RBPs, RNA binding proteins

## Abstract

The PIWI-interacting RNA (piRNA) pathway mainly consists of evolutionarily conserved protein factors. Intriguingly, many mutations of piRNA pathway factors lead to meiotic arrest during spermatogenesis. The majority of piRNA factor-knockout animals show arrested meiosis in spermatogenesis, and only a few show post-meiosis male germ cell arrest. It is still unclear whether the majority of piRNA factors expressed in spermatids are involved in long interspersed nuclear element-1 repression after meiosis, but future conditional knockout research is expected to resolve this. In addition, recent hamster knockout studies showed that a piRNA factor is necessary for oocytes—in complete contrast to the findings in mice. This species discrepancy allows researchers to reexamine the function of piRNA in female germ cells. This mini-review focuses on the current knowledge of protein factors derived from mammalian knockout studies and summarizes their roles in the biogenesis and function of piRNAs.

## Introduction

PIWI-interacting RNAs (piRNAs) are a distinct class of small RNAs [generally 24–31 nucleotides (nt) long] that are highly expressed in mouse testes. They are loaded onto PIWI proteins and function as an endogenous defense system against transposable elements (Aravin et al., 2004, 2006, 2007; Grivna et al., 2006; Kuramochi-Miyagawa et al., 2008). Some piRNAs are also involved in messenger RNA (mRNA) translation and mRNA/lncRNA elimination (Gou et al., 2014; Watanabe et al., 2015; Dai et al., 2019, 2020). Mice produce three types of germline piRNAs during spermatogenesis. Prenatal piRNAs first appear in the fetal testis and initiate transposon silencing *via* DNA methylation (Aravin et al., 2007, 2008; Carmell et al., 2007; Kuramochi-Miyagawa et al., 2008). The biogenesis of piRNAs in postnatal male germ cells is strikingly different from that in embryonic cells, as the majority of piRNAs are produced only by primary biogenesis after birth (Vourekas et al., 2012; Li et al., 2013). Postnatal piRNAs can be divided into pre-pachytene and pachytene piRNAs based on the timing of their expression and corresponding locus in the genome (Li et al., 2013). Because prenatal piRNA production and neonatal piRNA production involve continuous processes, they are rarely distinguishable during research. In most of the literature, prenatal piRNAs are classified as pre-pachytene piRNAs. Pachytene piRNAs are generally loaded onto MIWI (PIWIL1) or MILI (PIWIL2) (Vourekas et al., 2012; Li et al., 2013) and, unlike embryonic piRNAs, they have a strong 1U but no 10A bias, reflecting their primary biogenesis-dependent function (Vourekas et al., 2012; Li et al., 2013). These piRNA pathways are required during multiple stages of male germ cell development, including *de novo* DNA methylation, meiosis, and spermiogenesis (Kuramochi-Miyagawa et al., 2004; Carmell et al., 2007; Chuma and Nakano, 2013; Fu and Wang, 2014). In a review of piRNA pathway-knockout mice, meiosis arrest is described as the most common mouse phenotype and is mainly caused by abnormal piRNA production or retrotransposon DNA methylation in fetal testis (Yang and Wang, 2016).

Substantial past efforts have led to an understanding of piRNA biogenesis, which is thought to occur through either the primary or the secondary pathway. Primary piRNA biogenesis is coupled with a secondary piRNA amplification loop, the ping-pong cycle, in which piRNA pools, generated through primary processing, guide the MILI protein to slice transposon transcripts, providing substrates for piRNA generation and leading to the accelerated amplification of transposon-derived piRNAs (Brennecke et al., 2007; Gunawardane et al., 2007; Aravin et al., 2008). Primary piRNA biogenesis is initiated by the transcription of primary piRNA precursors derived from genomic regions called piRNA clusters—genomic regions mapped with a high density of piRNA sequences (Girard et al., 2006; Brennecke et al., 2007; Malone et al., 2009).

*De novo* DNA methylation occurs in prospermatogonia/gonocytes. During reprogramming, all DNA methylation marks are erased before being reset in germ cells, exposing the germline to essential challenge (Schaefer et al., 2007; Trasler, 2009). Loss of DNA methylation results in the activation of normally silenced transposable elements. Correct DNA methylation of transposons is vital for successful meiosis in male germ cells. Transposon demethylation was repeatedly observed in the testes of piRNA pathway mutants (Table 1), thus the pathway has been proposed to play a role in the *de novo* methylation of retrotransposons (Aravin et al., 2007; Carmell et al., 2007; Kuramochi-Miyagawa et al., 2008).

**TABLE 1:** Components of piRNA pathway in mice.

***Mus musculus***	***Mesocricetus auratus***	***Homo sapiens***	***Drosophila melano gaster***	**Spermatogenic arrest in KO mice**	**Expression pattern in male mice**	**Localization in male germ cell**	**Pre-pachytene piRNAs**	**Pachytene piRNAs**	**Transposon de-repression**	**References**
*Piwil2 (Mili)*	*Piwil2*	*PIWIL2*	*Aub*	Zygotene	E12.5 prosper matogonia to round spermatids	Cytoplasmic granules	√	√	LINE1 and IAP	Aravin et al., 2007; Aravin et al., 2008; De Fazio et al., 2011; Di Giacomo et al., 2013
*Mov10l1*	*Mov10l1*	*MOV10L1*	*Armi*	Zygotene	Gonocytes/type A spermatogonia to pachytene spermatocytes	Cytoplasmic granules	√	√	LINE1 and IAP	Frost et al., 2010; Vourekas et al., 2015; Zheng et al., 2010
*Pld6 (Mitopld)*	*Pld6*	*PLD6*	*Zuc*	Zygotene	E16.5 to the adult stage	N/A	√	N/A	LINE1	Huang et al., 2011; Ipsaro et al., 2012; Izumi et al., 2020; Nishimasu et al., 2012; Watanabe et al., 2011
*Tdrkh (Tdrd2)*	*Tdrkh*	*TDRKH*	*Papi*	Zygotene	Spermatogonia, spermatocytes, and round spermatids	Cytoplasmic granules	√	√	LINE1	Chen et al., 2009; Ding et al., 2019; Saxe et al., 2013
*Pnldc1*	*Pnldc1*	*PNLDC1*	*—*	Pachytene/spermatids	Spermatogonial stem cells to round sper matids in post natal testis; unknown in prenatal testis	N/A	√	√	LINE1	Bronkhorst and Ketting, 2018; Ding et al., 2017; Nishimura et al., 2018; Zhang et al., 2017b
*Tdrd1*	*Tdrd1*	*TDRD1*	*—*	Pachytene	Fetal prosper matogonia, postnatal spermatocytes and round spermatids	Cytoplasmic granules	N/A	√	LINE1	Chuma et al., 2003; Chuma et al., 2006; Reuter et al., 2009
*Asz1 (Gasz)*	*Asz1*	*ASZ1*	*Gasz*	Zygotene	Spermatogonia, spermatocytes, round spermatids	Cytoplasmic granules	√	√	LINE1	Ma et al., 2009; Zhang et al., 2016
*Mybl1 (A-myb)*	*Mybl1*	*MYBL1*	*—*	Pachytene	Mid-pachytene to round spermatids	Nuclei	N/A	√	N/A	Horvath et al., 2009; Li et al., 2013; Toscani et al., 1997
*Ddx4 (Mvh)*	*Ddx4*	*DDX4*	*vasa*	Zygotene	Male germ cells from E10.5 to round spermatids	Cytoplasmic granules	N/A	√	LINE1 and IAP	Kotaja and Sassone-Corsi, 2007; Kuramochi-Miyagawa et al., 2010; Noce et al., 2001; Saga, 2008; Siomi and Kuramochi-Miyagawa, 2009; Tanaka et al., 2000; Toyooka et al., 2000; Wenda et al., 2017
*Tdrd9*	*Tdrd9*	*TDRD9*	*Spn-E*	Zygotene	E13.5 prosper matogonia to round spermatids	Nucleus and cytoplasmic granules	N/A	N/A	LINE1	Shoji et al., 2009; Wenda et al., 2017
*Tdrd12*	*Tdrd12*	*TDRD12*	*BoYb*	Zygotene	From embryonic to the adult stages in mouse testes	N/A	√	N/A	LINE1 and IAP	Handler et al., 2011; Pandey et al., 2013; Yang et al., 2016
*Mael*	*Mael*	*MAEL*	*mael*	Pachytene	Spermatocytes and round and early elongating spermatids	XY body of spermatocytes and cytoplasmic granules of spermatids	N/A	√	LINE1 and IAP	Castañeda et al., 2014; Costa et al., 2006; Sato and Siomi, 2015
*Fkbp6*	*Fkbp6*	*FKBP6*	*Shu*	Pachytene	E12.5 prosper matogonia, cytoplasm and nucleus of spermatocytes	Cytosolic and not enriched in pi-bodies	√	N/A	LINE1	Crackower et al., 2003; Xiol et al., 2012
*Hsp90aa1*	*Hsp90aa1*	*HSP90aa1*	*Hsp83*	Meiotic arrest	E16.5 prosper matogonia to the adult stage	Cytosolic	√	N/A	LINE1	Grad et al., 2010; Ichiyanagi et al., 2014
*Gtsf1*	*Gtsf1*	*GTSF1*	*Gtsf1*	Meiotic arrest	E12.5 prosper matogonia to round spermatids	Cytoplasmic granules	√	N/A	LINE1 and IAP	Dönertas et al., 2013; Ohtani et al., 2013; Yoshimura et al., 2009; Yoshimura et al., 2018
*Spocd1*	*Spocd1*	*SPOCD1*	*pps*	Pachytene	E14.5 to PN1 prosper matogonia	Nucleus and cytoplasmic granules	×	N/A	LINE1 and IAP	Zoch et al., 2020
*Tex15*	*Tex15*	*TEX15*	*—*	Zygotene	Transcript abundance was high at E16.5 and increased at PN2.5	N/A	×	N/A	LINE1 and IAP	Schöpp et al., 2020; Yang et al., 2008; Yang et al., 2020
*Uhrf1*	*Uhrf1*	*UHRF1*	*—*	Pachytene	E15.5 prosper matogonia to round spermatids	Nuclei of neonatal prospermatonia at PN0, spermatogonia, late pachytene spermatocytes, and early round spermatids; cytoplasm of fetal prosper matogonia at E15.5, pre-leptotene, leptotene, zygotene, and early pachytene spermatocytes	N/A	√	LINE1	Dong et al., 2019
*Tut4/7*	*Tut4/7*	*TUT4/7*	*—*	Pachytene	Spermatogonia to round spermatids	Cytoplasmic granules	N/A	√	LINE1	Morgan et al., 2019
*Piwil1 (Miwi)*	*Piwil1*	*PIWIL1*	*Aub*	Round spermatid	Pachytene spermatocytes to elongating spermatids	Cytoplasmic granules	×	√	LINE1	Carrieri et al., 2017; Dai et al., 2019; Dai et al., 2020; Deng and Lin, 2002; Gou et al., 2014; Gou et al., 2017; Li et al., 2020; Oud et al., 2021; Reuter et al., 2011; Zhao et al., 2013
*Tdrd5*	*Tdrd5*	*TDRD5*	*qin*	Round spermatid/meiotic prophase	E7.25 PGCs to round-spermatid stage	Spots in the nucleus and cytoplasmic granules	N/A	√	LINE1	Ding et al., 2018; Smith et al., 2004; Yabuta et al., 2011
*Henmt1*	*Henmt1*	*HENMT1*	*Hen1*	Spermatids	Spermatogonia to elongated spermatids	Cytoplasmic granules	√	√	LINE1 and IAP	Kirino and Mourelatos, 2007; Lim et al., 2015
*Ythdc2*	*Ythdc2*	*YTHDC2*	*Bgcn*	Zygotene	YTHDC2 expression during the first wave of spermatogenesis.	Cytoplasmic granules	N/A	√	N/A	Bailey et al., 2017
*Piwil4 (Miwi2)*	*Piwil4*	*PIWIL4*	*Piwi*	Zygotene	E15.5 to PN1 prosper matogonia	Nucleus and cytoplasmic granules	N/A	N/A	LINE1 and IAP	Carmell et al., 2007; Carrieri et al., 2017; Zoch et al., 2020

PIWI-interacting RNA pathway consists of many evolutionarily conserved protein factors. This mini-review focuses on our current knowledge of protein factors in mammals by summarizing their roles in the biogenesis and function of piRNAs based on research with gene-knockout models.

## Primary piRNA Biogenesis

Primary piRNA biogenesis is a stepwise process that starts with the transcription of long single-stranded precursor transcripts. A-MYB, which is the only transcription factor known to be involved in transcriptional regulation of pachytene piRNA precursor, also regulates the transcription of many pachytene piRNA pathway genes (Li et al., 2013). Through its ATP-dependent RNA helicase activity, MOV10L1 selectively binds to piRNA precursor transcripts and feeds them to MitoPLD, which catalyzes the first cleavage step of piRNA processing to generate piRNA intermediates. MOV10L1 is associated with MILI, MIWI, and MIWI2 (PIWIL4) in mouse testes; its expression emerges in prenatal gonocytes, peaks in pachytene spermatocytes, and ceases in post-meiotic spermatids. Disruption of *Mov10l1* results in defects in both the transcriptional and posttranscriptional de-repression of transposons, consistent with the lack of retrotransposon-derived pre-pachytene piRNAs in *Mov10l1* mutant testis (Zheng et al., 2010). Primary spermatocytes of *Mov10l1*^–/–^ mice show the activation of long terminal repeat-containing retrotransposons and long interspersed nuclear element-1 (LINE1) retrotransposons, followed by cell death, causing infertility in males and the complete blockage of spermatogenesis at the zygotene stage of meiosis I prophase (Frost et al., 2010; Zheng et al., 2010; Vourekas et al., 2015).

MitoPLD is localized on the surface of the mitochondrial outer membrane in mouse germlines (Choi et al., 2006; Watanabe et al., 2011) and is a candidate for the nuclease that generates piRNA intermediates. In MitoPLD-mutant mouse testes, both primary and secondary piRNAs were significantly decreased, and piRNA biogenesis disruption was accompanied by a spike in LINE1 retrotransposon expression and genomic demethylation. MitoPLD-knockout mice showed arrested spermatogenesis at the meiosis zygotene stage (Huang et al., 2011; Watanabe et al., 2011), and MitoPLD has endoribonuclease activity on single-stranded RNAs *in vitro* (Ipsaro et al., 2012). A recent *Bombyx mori* study found that Zucchini (homolog of MitoPLD) requires Armi, GPAT1, and Gasz to cleave Siwi-loaded pre-pre-piRNAs *in vitro* (Izumi et al., 2020). In addition, the N6-methyadenosine (m6A) reader, YTHDC2, binds to specific piRNA precursors. P12 *Ythdc2*^–/–^ mice exhibited much lower pachytene piRNA precursor levels than normal (Bailey et al., 2017).

MILI is one of three mouse homologs of the PIWI family that are defined by their conserved PAZ and Piwi domains. MILI, an important mediator of sense piRNA processing from retrotransposons and other cellular transcripts (Kuramochi-Miyagawa et al., 2004; Aravin et al., 2008), is expressed in the cytoplasm of testicular germline stem cells, spermatogonia, and early spermatocytes. In a mouse MILI-null mutant, spermatogenesis was completely blocked at the prophase of meiosis I from the zygotene to early pachytene (Kuramochi-Miyagawa et al., 2004). Acting as a piRNA-guided endonuclease, MILI initiates secondary piRNA biogenesis, which is vital for LINE1 and Intracisternal A particle (IAP) silencing (Aravin et al., 2007; De Fazio et al., 2011). Functions of MILI beyond piRNA biogenesis have been described recently. MILI forms a stable and RNA-independent complex with eIF3a and is associated with the eIF4E- and eIF4G-containing 5′-end 7-methylguanosine (m7G) cap-binding complex, which may positively regulate the translation of genes essential for germline stem cell self-renewal and differentiation (Unhavaithaya et al., 2009).

TDRKH, another mitochondria-anchored protein involved in primary piRNA biogenesis (Saxe et al., 2013), is a Tudor family protein that contains evolutionarily conserved Tudor and KH domains (Zhang et al., 2017a); it controls the entire MIWI/MIWI2-bound piRNA population and enables the trimming of MILI-bound piRNAs. *Tdrkh* mutants display meiotic arrest at the zygotene stage, with loss of DNA methylation of LINE1 retrotransposons and consequential retrotransposon de-repression (Saxe et al., 2013; Ding et al., 2019). Associated with MIWI and MIWI2 *via* the binding of symmetrically dimethylated arginine (sDMA), TDRKH is the scaffold for interactions between PIWI–piRNA complexes and PNLDC1. The exonuclease trims the 3′-end of piRNA intermediates to their mature length (Ding et al., 2017; Zhang et al., 2017b; Bronkhorst and Ketting, 2018; Nishimura et al., 2018). The 3′-end of mature piRNA is 2′-*O*-methylated by HENMT1, yet correct 3′ truncation is not necessary for 3′-end 2′-*O*-methylation (Yang et al., 2006; Zhai and Meyers, 2012; Peng et al., 2018). In addition, TUT4/7 mediates the 3′ uridylation of 30- to 31-nt-long piRNAs, but its effect is unknown (Morgan et al., 2019).

## Secondary piRNA Biogenesis

**FIGURE 1:**
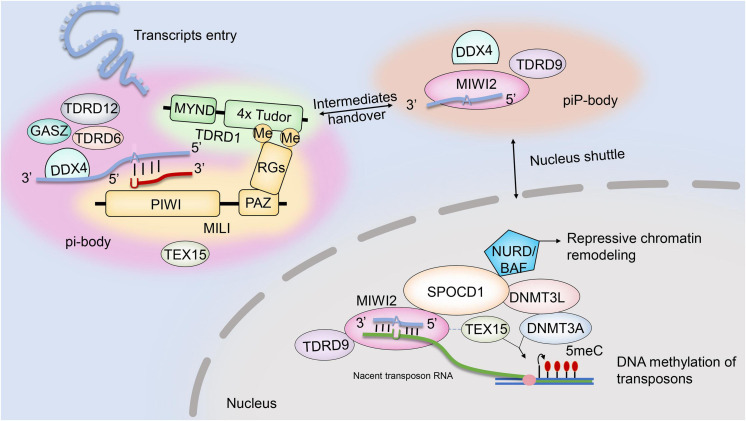
Ping-pong cycle and MIWI2-piRNA-guided chromatin remodeling.

The piRNA pathway mediation of transposon posttranscriptional silencing is regulated by interactions between two RNA–protein complexes: pi-body and piP-body. While MILI–piRNA and MIWI2–piRNA complexes are key to the assembly and function of pi-body and piP-body, several other factors are also believed to be required. The existing evidence for the possible interactions and localizations of those factors is summarized in this review (Figure 1). Another member of the Piwi protein family, MIWI2, is coexpressed with MILI during embryonic testis development. Once loaded with secondary piRNAs, MIWI2 is shuttled from the cytoplasm to the nucleus to mediate repressive chromatin remodeling mainly *via* the promoter regions of transposons. However, it remains unclear whether MIWI2–piRNA complexes regulate the methylation patterns of other genomic regions (Schöpp et al., 2020). Loss of MIWI2 function affects the DNA methylation of LINE1 elements. Miwi2-deficient mice displayed zygotene-stage meiotic arrest, defective synapsis and double-strand break repair, and progressive loss of germ cells with age (Carmell et al., 2007).

TDRD9, a TDRD family member, was also investigated as an essential partner of MIWI2. TDRD9 complexes with MIWI2 through its Tudor domain, which binds to sDMA sites of MIWI2. TDRD9 is expressed in the cytoplasm and nucleus of embryonic prospermatogonia, mitotic spermatogonia, meiotic spermatocytes, and haploid spermatids in the testis (Shoji et al., 2009). Abolishing TDRD9 expression caused male mouse sterility and meiotic arrest at the zygotene stage: the spermatogenic cells faithfully initiated meiotic DNA recombination, but homologous chromosomes failed to undergo synapsis (Shoji et al., 2009). TDRD9 participates in the biogenesis of secondary piRNAs by ensuring the proper selection of *Line1* sequences for the ping-pong amplification loop (Shoji et al., 2009). Although dispensable for piRNA biogenesis, TDRD9 ATPase activity is indispensable for its nuclear localization and transcriptional silencing of transposable elements (Wenda et al., 2017).

Other TDRD family members, including TDRD1 and TDRD12, interact with MILI in the ping-pong cycle. TDRD1 recognizes arginine dimethylation in MILI (Chuma et al., 2003, 2006) and may regulate the entry of transcripts into piRNA biogenesis pathways. The loss of TDRD1 does not affect the abundance of MILI-bond piRNA but rather its constituents: ribosomal RNA- and genic-derived piRNA proportions increase, and transposon-derived piRNAs in MILI ribonucleoprotein (RNP) populations change substantially. In addition, the correct nuclear localization of Miwi2 needed for LINE1 transposon methylation was almost lost, and LINE1 transposons were repressed as consequences of TDRD1 knockout (Reuter et al., 2009). TDRD1 also draws ping-pong cycle factors together to promote their activity. DDX4 and FKBP6, components of the TDRD1 protein complex, are required for the loading of MIWI2-bound secondary piRNAs. FKBP6 may recruit HSP90AA1 for the loading of secondary piRNA intermediates onto MIWI2 (Xiol et al., 2012).

TDRD12 forms complexes with MILI piRNP in an RNA-dependent manner and is associated with TDRD1. TDRD12 might facilitate the RNP remodeling required for the inter-Piwi (MILI and MIWI2) exchange of piRNA intermediates essential for the biogenesis of MIWI2 piRNAs (Pandey et al., 2013). The biogenesis of piRNAs that associate with MILI appeared normal, with unchanged genome annotation profiles, in mice lacking TDRD12; however, MIWI2-bond piRNA biogenesis was almost absent. When TDRD12 was deficient, spermatogenesis stalled in the zygotene–pachytene transition stage of meiosis.

DDX4, which is expressed in the cytoplasm of various male germ cells (E10.5 to round spermatids) (Kuramochi-Miyagawa et al., 2010), has RNA helicase activity (Sengoku et al., 2006) and N terminal sDMAs characterized by Tudor domains (Kirino et al., 2010). Multiple mouse models have been adopted to investigate the roles of DDX4 in spermatogenesis and piRNA pathways. DDX4-knockout mice exhibited complete spermatogenic arrest at the zygotene stage, and mutation of the RNA-helicase domain of DDX4 (DDX4 was expressed normally but catalytically dead) also disrupted spermatogenesis. *Ddx4*^–/catalytically dead^ mouse spermatogenesis did not proceed beyond meiotic pachytene in spermatocytes, while germ cells in *Ddx4*^+/catalytically^ ^dead^ mice completed meiosis but uniformly arrested during the development of round spermatids (Wenda et al., 2017). The essential role of DDX4 in the piRNA pathway was recently revealed: DDX4 is required for RNP remodeling during the loading of secondary piRNA intermediates onto MIWI2. The endonucleolytic cleavage of a target transcript by cytosolic MILI generates a piRNA precursor, which is processed into phased pre-piRNA intermediates (Han et al., 2015; Mohn et al., 2015; Yang et al., 2016). Mice lacking catalytically active DDX4 were still able to generate MILI slicer products but failed to transfer pre-piRNA intermediates to the ping-pong biogenesis machinery. Therefore, no MIWI2-bound piRNA was detected in mice with catalytically dead DDX4, and MIWI2 failed to maintain the necessary DNA methylation of L1 retrotransposons (Wenda et al., 2017). Furthermore, catalytically dead DDX4 also trapped MILI and MIWI, pachytene piRNAs, and slicer products of transposon and genic mRNAs, suggesting it functions in posttranscriptional regulation in post-meiotic stages (Wenda et al., 2017). In addition, reduced GTSF1 protein, which co-localizes with TDRD9 and MIWI2 in piP-bodies, resulted in target RNA remaining unsliced at the cleavage site for MILI-directed secondary piRNA processing (Yoshimura et al., 2018).

## Transposable Element Methylation by PIWI Pathway

*Mael* is highly expressed in mouse testes, and the protein’s location alternates throughout spermatogenesis. MAEL, found in the cytoplasm in spermatocytes and shuttled to the nucleus in spermatids (Soper et al., 2008; Pandey and Pillai, 2014), comprises a high-mobility group box and a MAEL domain that is predicted to adopt an RNase H-like fold. Meiotic entry was delayed in *Mael*-null spermatogenic cells (Soper et al., 2008). Although *Mael*-knockout mice phenocopied *Mili-* and *Miwi2*-knockout mice, pre-pachytene arrest was intact in *Mael-*null testes. MAEL is speculated to function in post-piRNA production steps by facilitating the nucleo-cytoplasmic trafficking of MIWI2–piRNA complexes (Soper et al., 2008; Pandey and Pillai, 2014). In post-meiotic spermatogenesis, MAEL interacts with MILI, MIWI, and TDRD6, binding pachytene piRNA precursors and enabling piRNA intermediate processing (Pandey and Pillai, 2014; Sato and Siomi, 2015).

A recent study revealed that TEX15, a nuclear protein, is an essential partner of MIWI2 in piRNA-directed *de novo* methylation and silencing of transposable elements in fetal gonocytes (Schöpp et al., 2020). TEX15 contains a DUF3715 domain, which is also found in other TE-silencing proteins (Tchasovnikarova et al., 2015; Liu et al., 2018). In *TEX15*-null spermatocytes, SPO11-mediated DSB formation was normal, but DSB repair was absent because of a failure in the DMC1 assembly, resulting in zygotene-stage meiotic arrest (Yang et al., 2008). Although TEX15 interacts with MILI in the cytoplasm, it is not required for primary or secondary piRNA biogenesis in mouse gonocytes. TEX15 also interacts with MIWI2 in the nucleus in an RNA/DNA-dependent manner, yet the nuclear localization of MIWI2 remains unchanged in *TEX15*-null gonocytes. Considering that loss of TEX15 causes demethylation in LINE1 and IAP transposon promoter regions, it may be a predominant nuclear executor of TE *de novo* methylation downstream of piRNA pathways (Schöpp et al., 2020; Yang et al., 2020).

SPOCD1, another MIWI2 interactome member, facilitates MIWI2 activity in the nucleus. *Spocd1*-null spermatocytes undergo early-pachytene-stage meiotic arrest, but both primary and secondary piRNA biogeneses remain. Loss of IAP and LINE1 transposon *de novo* DNA methylation and consequential transposon de-repression were observed in *Spocd1*-knockout testes. SPOCD1 engages with MIWI2 in an RNA/DNA-dependent manner and facilitates MIWI2 nuclear activity by summoning chromatin remodeling and DNA methylation machinery to the promoters of transcribing transposons (Zoch et al., 2020). SPOCD1 contains a SPOC domain, which was previously found to recruit transcriptional repressors (Ariyoshi and Schwabe, 2003; Mikami et al., 2014), and a nuclear localization signal. SPOCD1 co-immunoprecipitated with DNMT3L and DNMT3A, components of the *de novo* methylation machinery and the NURD (Kloet et al., 2015) and BAF (Mashtalir et al., 2018) repressive chromatin remodeling complexes.

UHRF1 maintains the crosstalk between the PIWI pathway and repressive chromatin remodeling machinery. UHRF1 was found to be abundant in the nuclei of neonatal prospermatonia at P0, as well as spermatogonia, late pachytene spermatocytes, and early round spermatids, and shifted into the cytoplasm of fetal prospermatogonia during spermatocyte E15.5, pre-leptotene, leptotene, zygotene, and early pachytene. The conditional deletion of *Uhrf1* in differentiating spermatogonia led to pachytene-stage meiotic arrest. UHRF1 interacts with PRMT5 (Kirino et al., 2009; Zhao et al., 2009; Wang et al., 2015), an arginine methyltransferase, to regulate repressive histone arginine modifications (H4R3me2s and H3R2me2s) (Ancelin et al., 2006; Migliori et al., 2012) and piRNA biogenesis by controlling the localization of PIWI pathway proteins (MILI, MIWI, and TDRKH). UHRF1 depletion also induces global loss of DNA methylation during spermatogenesis. UHRF1 appears to play essential roles in the crosstalk between the piRNA pathway and repressive epigenetic pathways, providing new clues to piRNA pathway functions (Dong et al., 2019).

## Repression of LINE1 Retrotransposons in Germ Cells

LINE1 retrotransposons are members of the most abundant class of transposable elements in mammals, accounting for ∼20% of mouse and human genomes. Up to 3,000 and 100 copies of LINE1 are intact and active in mice (Deberardinis et al., 1998) and humans (Sassaman et al., 1997; Mandal and Kazazian, 2008), respectively. In male piRNA pathway mutants, LINE1 activated late embryonic germ cells or early and mid-pachytene spermatocytes (Yang and Wang, 2016). Most male mouse piRNA pathway mutants exhibit meiotic arrest and sterility, but this effect is not observed in females (Yang and Wang, 2016). Notably, LINE1 de-repression in spermatocytes does not necessarily lead to meiotic arrest, such as in *Henmt1-*knockout animals (Lim et al., 2015). Some mouse mutants of *Miwi* (Deng and Lin, 2002), *Pnldc1* (Ding et al., 2017; Zhang et al., 2017c; Bronkhorst and Ketting, 2018; Nishimura et al., 2018), *Tdrd5* (Yabuta et al., 2011; Ding et al., 2018), and *Henmt1* (Lim et al., 2015), etc., still produce post-meiotic germ cells. Interestingly, although a large proportion of MIWI-piRNAs were thought to originate from non-transposon-related regions (Vourekas et al., 2012), LINE1 de-repression was found in *Miwi*-knockout mouse spermatids (Reuter et al., 2011). MIWI slicer activity involved in the direct cleavage of transposon mRNAs in spermatids (Reuter et al., 2011) is also chromatoid body location dependent but may not be piRNA dependent (Ding et al., 2019). In a *Pnldc1* mutant, dramatically reduced MIWI protein and MIWI-piRNAs, without spermatid LINE1 de-repression, were seen (Ding et al., 2017; Zhang et al., 2017c; Nishimura et al., 2018), and the remaining MIWI in the mutant possibly played a role in LINE1 repression (Ding et al., 2019). Spermatids in *Henmt1*-knockout mice also showed activated LINE1 that was unassociated with MIWI slicer activity (Lim et al., 2015). These results suggest that LINE1 repression also occurs in spermatids. Most piRNA factor knockouts display meiotic arrest; therefore, there is a lack of information on LINE1 repression after meiosis. Pachytene piRNA cluster is usually non-repeat origin, thus the mechanism of LINE1 repression after meiosis needs further exploration. The active LINE1 ORF1p is often found in the cytoplasm of spermatocytes but is more commonly seen in round spermatid nuclei, although the reason for this is unknown. A recent conditional knockout (cKO) study provided examples of how this process can be explored; Tdrkh^cKO^ driven by Stra8-Cre, but not Mov10l1^cKO^, showed obvious LINE1 de-repression in spermatids (Ding et al., 2019). This raises questions about whether piRNA factor genes expressed in spermatids, such as *Tdrd1*, *Asz1*, *Mybl1*, *Ddx4*, *Tdrd9*, *Mael*, *Gtsf1*, *Uhrf1*, *Tut4/7*, and *Tdrd5*, are involved in LINE1 inhibition after meiosis (Table 1).

Because of knockout mouse studies, piRNA pathway is believed to be unnecessary in mammalian female germ cells (Yang and Wang, 2016). In mouse oocytes, the ribonuclease MARF1, which is not associated with piRNA, is considered to be involved in LINE1 inhibition in oocytes (Su et al., 2012a,b; Yao et al., 2018). This phenomenon suggests that a transposon inhibition system other than piRNA may function in mouse oocytes. Apart from mice, most mammals have four PIWI genes. PIWIL3, which is not expressed in mice, binds to a class of piRNAs of 19 and 20 nt in hamster and human oocytes, respectively (Yang et al., 2019; Ishino et al., 2021). *PIWIL3*-deficient female hamsters have reduced fertility (Hasuwa et al., 2021). Furthermore, abolishing piRNA factors *PIWIL1*, *PLD6*, and *MOV10L1* in golden hamsters led to female infertility, with embryos arresting at the two-cell stage (Ishino et al., 2021; Zhang et al., 2021). Therefore, the function of piRNA in oocytes may be significantly different among mammalian species.

## Conclusion

Previous studies using knockout mice have revealed the formation of piRNA in mammals and its role in male germ cells. Most piRNA factor knockouts showed spermatogenesis arrest in meiosis, but a few showed male germ cell arrest after meiosis. It is unclear whether the majority of piRNA factors expressed in spermatids are involved in LINE1 repression after meiosis, and future cKO research is required. In addition, in recent hamster gene-knockout studies, a piRNA factor was found to be necessary for oocytes, a complete contrast to findings in mice. This species difference allows researchers to reexamine the function of piRNA in female germ cells, which should broaden our knowledge on female infertility in humans.

## Author Contributions

ML, YZ, and YL: conceptualization. YL and YZ: literature search. ML, YZ, and YL: writing—original draft preparation. ML and YL: writing—review and editing. YL: visualization of histological structures. All authors read and approved the final version of manuscript.

## Conflict of Interest

The authors declare that the research was conducted in the absence of any commercial or financial relationships that could be construed as a potential conflict of interest.
